# First DNA Barcode Reference Library for the Identification of South American Freshwater Fish from the Lower Paraná River

**DOI:** 10.1371/journal.pone.0157419

**Published:** 2016-07-21

**Authors:** Juan Díaz, Gabriela Vanina Villanova, Florencia Brancolini, Felipe del Pazo, Victoria Maria Posner, Alexis Grimberg, Silvia Eda Arranz

**Affiliations:** 1 Instituto de Biología Molecular y Celular de Rosario (IBR, CONICET-UNR), Rosario, Santa Fe, Argentina; 2 Area Biología, Facultad de Ciencias Bioquímicas y Farmacéuticas, Universidad Nacional de Rosario, Rosario, Santa Fe, Argentina; 3 Instituto de Limnología “Dr. Raúl A. Ringuelet” (ILPLA, CONICET-UNLP), La Plata, Buenos Aires, Argentina; 4 Laboratorio de Biotecnología Acuática (FCByF-UNR/SeCTEI-Santa Fe), Acuario del río Paraná, Rosario, Santa Fe, Argentina; University of Minnesota, UNITED STATES

## Abstract

Valid fish species identification is essential for biodiversity conservation and fisheries management. Here, we provide a sequence reference library based on mitochondrial cytochrome c oxidase subunit I for a valid identification of 79 freshwater fish species from the Lower Paraná River. Neighbour-joining analysis based on K2P genetic distances formed non-overlapping clusters for almost all species with a ≥99% bootstrap support each. Identification was successful for 97.8% of species as the minimum genetic distance to the nearest neighbour exceeded the maximum intraspecific distance in all these cases. A barcoding gap of 2.5% was apparent for the whole data set with the exception of four cases. Within-species distances ranged from 0.00% to 7.59%, while interspecific distances varied between 4.06% and 19.98%, without considering *Odontesthes* species with a minimum genetic distance of 0%. Sequence library validation was performed by applying BOLDs BIN analysis tool, Poisson Tree Processes model and Automatic Barcode Gap Discovery, along with a reliable taxonomic assignment by experts. Exhaustive revision of vouchers was performed when a conflicting assignment was detected after sequence analysis and BIN discordance evaluation. Thus, the sequence library presented here can be confidently used as a benchmark for identification of half of the fish species recorded for the Lower Paraná River.

## Introduction

Reliable species identification is necessary for conservation and sustainable exploitation of natural resources. DNA sequences of highly conserved genes have been used as a tool to identify biological species. This approach became especially relevant when identification based on morphological characters is not possible (e.g. larvae, eggs and fragmented tissue). A short (~650-bp) DNA fragment from the mitochondrial 5' end region of the cytochrome c oxidase subunit I (COI) gene has been extensively used as a universal standard DNA barcode for metazoan species identification [1]. A reference COI sequence library derived from expert-identified reference material is the first step to further assign organisms into species by matching the sequence of an unknown sample to the reference library. DNA barcoding standardized methodology [2] relies on the assumption that inter-species genetic variation is greater than the intra-species variation (“DNA barcoding gap”) [3]. Moreover, variability in a molecular marker often opened the door to the discovery of new species [4].

The taxonomic reliability of generated DNA barcodes must be exhaustively verified previous to the construction of a reference COI sequence library. Different clustering methods for species-specific assignment using molecular data are available to analyse sequence divergence of the COI barcoding region [5–7] as well as to test whether specimens assigned to a species can be found within the same group.

The DNA barcoding approach has been proven to discriminate a high percentage of fish species from freshwater habitats [8, 9] including the recently radiated Neotropical ichthyofauna from the Upper Paraná River basin [10]. Furthermore, application of DNA barcoding revealed cryptic fish species across the Brazilian Amazon [11].

The Paraná/Paraguay system in South America has the tenth highest discharge among the largest rivers in the world [12], and has one of the richest and most diverse fish fauna [13]. The Paraná River travels 3998 km southwards, from its sources in the Precambrian Brazilian Shield to its mouth in the Río de la Plata estuary (35° S). The Upper Paraná has been historically separated from the rest of the basin by the Guaíra Falls and, later on, by the functional barrier of the Itaipú Dam (25°35’31” S; 54°35’32” W), generating a distinct ichthyofaunistic province [14]. The Lower Paraná River is an un-dammed freshwater axis that extends from the confluence of the Paraguay and Paraná Rivers (Km 1244) to the Río de la Plata (Km 0) estuary [15]. In its lower section (32°04’11.41” S– 60°38’17.54” O), the Paraná River divides its flow forming a delta covering 3500 km2 [15]. The variety of habitats is remarkable, including lagoons, streams, wetlands and floodplains [16], offering a variety of feeding, mating, spawning and nursery grounds [17] for different fish species. At least 185 fish species [18], representing most Lower Paraná River fish species described [19], are present in the area, including 8 endemic and 2 exotic species. In addition, the Paraná Delta functions as a migratory exchange route for anadromous fishes, such as bagre marino (*Genidens barbus*) and pejerrey (*Odontesthes sp*.), which run up the river from the Atlantic Ocean and potadromous fish species of freshwater brassylic-tropical lineage which reach the Río de la Plata estuary [20], such as sábalo (*Prochilodus lineatus*) and dorado (*Salminus brasiliensis*) [21]. Twelve fish species are commercially exploited for either domestic consumption or export [22]. Examples are: sábalo (*P*. *lineatus*) which is the main component of fish catches in the Lower Paraná River [23] and surubí (*Pseudoplatystoma corruscans*). Catch decreases have been observed in the past decades [24] in species such as pacú (*Piaractus mesopotamicus*), manguruyú (*Paulicea luetkeni*), surubí (*Pseudoplatystoma sp*.*)*, salmón de río (*Brycon orbignyanus*), anchoa (*Lycengraulis grossidens*) and pejerrey (*Odontesthes sp*.), all of them with great sport and commercial value.

Similar to other wetlands, the Paraná Delta is strongly influenced by human activities such as extensive agriculture, cattle rising, commercial fishing and commercial transportation [25], which represent great threats to local biodiversity. Although human activities negatively influence fish biodiversity, distortion of the flood pulse associated to climate change could be another cause for the loss in fish biodiversity in this river section [26].

Records of fish species identification through DNA barcoding in the Lower Paraná River were not available until this study. Only 36 freshwater fish species belonging to Salado River lakes- Pampa Plain have been identified through DNA barcode in Argentina [27].

Given the great power of DNA barcoding to identify fish species and considering the unique assemblage of Lower Paraná River fish along with future changing scenarios, the present study aimed to group the first comprehensive reference COI sequence library for fishes of this region. Also, to test the effectiveness of the barcoding methodology for their identification in future studies.

## Materials and Methods

### Specimen collection and sampling area

Several fishing techniques were used for sampling. Sampling methods included gill nets, lift nets, slat traps, hoop nets and angling. Animals were handled with maximum care to prevent or minimize injuries during studies. All sampling procedures and methods were in accordance with the FishBol international project recommendations and the Guidelines for the use of fishes in research by the American Fisheries Society.

**Fig 1:**
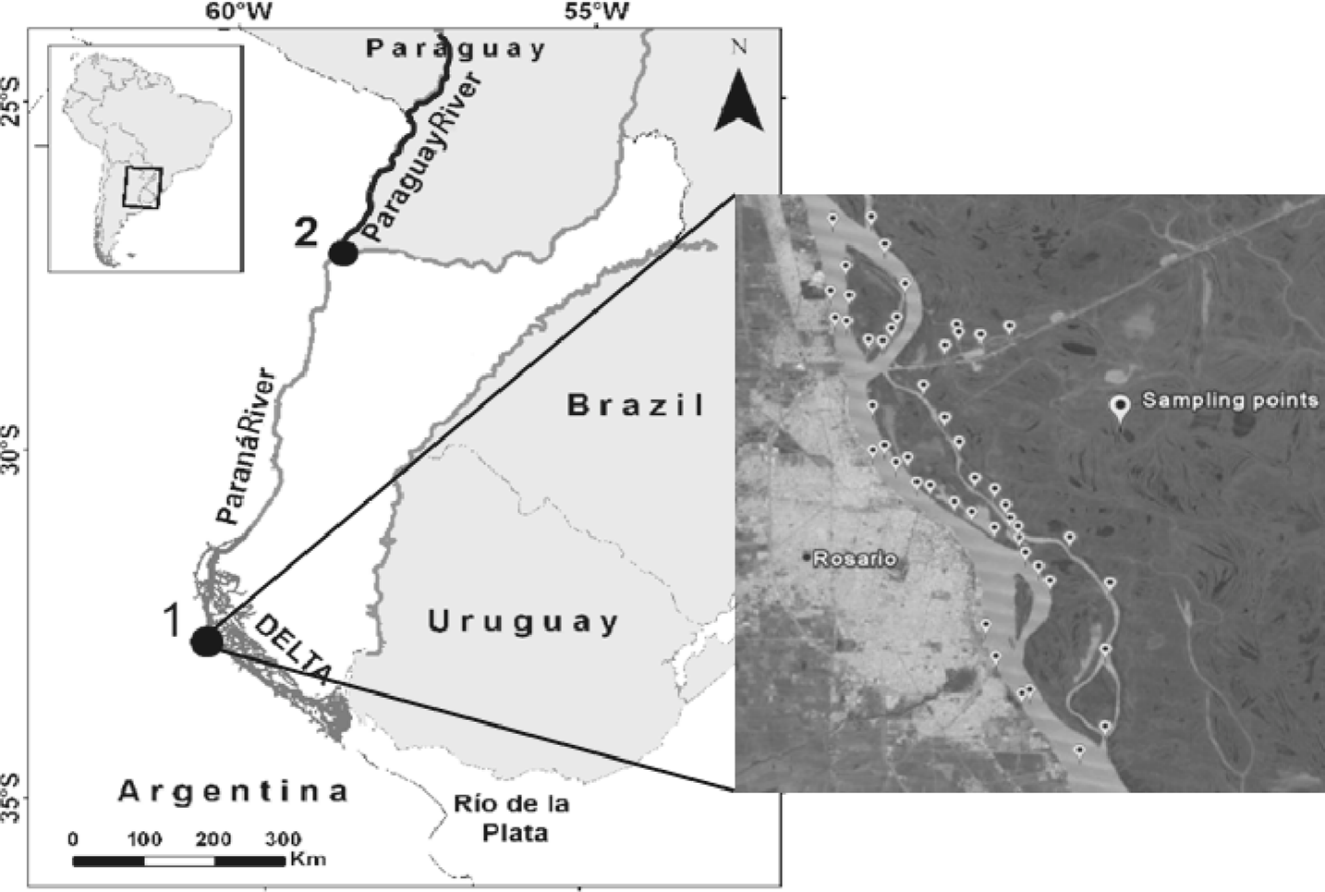
Sample sites map. Map of the Lower Parana River Basin showing the 50 sampling points at sites 1 and 2.

A total of 308 specimens were sampled between 2012 and 2013 from a wide area of the Lower Paraná River near the city of Rosario (50 sampling points, site 1) with the exception of 8 specimens obtained at one sampling point at site 2 (Fig 1). The locations involved in the study were not part of any protected area, reserve forests or national parks. The General Direction of Natural Resources of the Production Ministry from Entre Rios Province in compliance with the law N° 4892/70, issued the permission to conduct this study in Paraná River and Delta from Diamante to Gualeguay city. No specific permissions were required for sampling point 2 since tissue samples and photograph of Specimens were obtained from sport and commercial fishermen. The selected area encompasses a variety of environments, such as lagoons, streams, wetlands, the main river channel and its coasts. Collected specimens were anesthetized by immersion in 1% benzocaine in water and euthanized by benzocaine excess. A small piece (5–7 mm³) of muscle or fin tissue was removed from the right side of each fresh fish and preserved in 96% ethanol at -20°C. Specimens were photographed, labeled and fixed in 10% formaldehyde solution for 7 days. Occasionally, tissues were collected through the support of fisherman. Tissues and vouchers specimens were stored in 96% ethanol and deposited in the Fish Collection of the Angel Gallardo Natural Sciences Provincial Museum, Rosario, Argentina. Some tissue samples lack morphological vouchers, but have a “photographic voucher” according to the Fish-BOL collaborator’s protocol [28]. All specimens for this study were obtained in compliance with animal welfare laws, local guidelines and national policy in the realm of the Argentine Republic.

### Fish identification

Taxonomic determination was performed following the identification reliability levels 1 and 2 according to the Fish-BOL collaborator’s protocol [28]. Fish identification was determined to the species level whenever possible; using the following references [29–47]. Fish classification follows Eschmeyer (2014) [48].

### Extraction, PCR amplification, and DNA sequencing

Total genomic DNA was extracted from muscle or fin tissue from each specimen using an automated Glass Fiber protocol [49]. A 648-bp DNA sequence from the 5’ region of COI [50] was subsequently amplified by PCR, with the following thermal cycling: initial denaturation at 95°C for 2 min, 35 cycles at 94°C for 30 s, 52°C for 30 s, and 72°C for 1min, with a final extension step at 72°C for 10 min. The 12.5 μl PCR reaction mixes included 6.25 μl of 10% trehalose, 2 μl of ultrapure water, 1.25 μl 10X PCR buffer [200 mMTris-HCl (pH 8.4), 500 mMKCl], 0.625 μl MgCl_2_ (50 mM), 0.125 μl of C_FishF1t1 and C_FishR1t1 primer combination (0.01 mM) [51], 0.062 μl of each dNTP (10 mM), 0.060 μl of Platinum^®^Taq Polymerase (Invitrogen), and 2 μl of DNA template. PCR products were sent to the Biodiversity Institute of Ontario facility for sequencing on an ABI 3730 capillary sequencer (Applied Biosystems, Inc.) following the manufacturer’s instructions.

### Sequence alignment and data analysis

Bidirectional sequences were assembled in SEQSCAPE version 2.1.1 (Applied Biosystems, Foster City, CA, USA), and manually edited. Assembled DNA sequences were submitted to GenBank (accession numbers: KU288760-KU289067).

The COI assemble sequences were analyzed using Alignment Browser and Sequence Composition tools, both available in the Sequence Analysis Module of BOLD. Genetic distances among and within species were estimated using MEGA, version 6.0 [52]. The applied model of nucleotide substitution was estimated using the best fit substitution model tool [53] available in the same software. General time reversible substitution model (GTR) with a Gamma distribution of variable sites and invariable sites was the model that best fit the substitution pattern of the dataset with ti/tv = 3.48, α = 0.83, and I = 0.56.

Genetic distances among and within species were also estimated using the Kimura two-parameter (K2P) substitution model [54], implemented in the Distance Summary tool in BOLD. This is the standard model for DNA barcoding data sets and one of the most commonly used models to describe distance between species using COI. Since no significant differences were found in estimated distances and tree topologies between GTR and K2P models, the latter was chosen for comparison purposes. Haplotype identification was performed using DnaSP 5.10.01 [55].

Neighbour Joining (NJ) and Maximum Likelihood (ML) trees based on K2P genetic distance were created to provide a graphic representation for the patterning of distance between species using the MEGA 6 software [52]. Node robustness was inferred with 1000 bootstrap replicates. Comparisons at the species level of the maximum intraspecific genetic distance with the minimum distance to the nearest neighbour were performed applying the BOLD’s ‘Barcoding Gap Analysis’ tool.

Three different clustering methods, Barcode Index Number (BIN) system [5], Poisson Tree Processes (PTP) model [6] and Automatic Barcode Gap Discovery (ABGD) [7], were used to confirm the concordance between sequence clusters and species designations by taxonomy. These methods were selected based on their general popularity and strong performance in previous studies [56,57]. The three methods clustered COI sequence data into operational taxonomic units (OTUs) independent of prior taxonomic assignment. PTP reports were generated with default settings using the ML solution. ABGD clustering was carried out using the K2P distance model applying the following parameters: Pmin = 0.001, Pmax = 0.1; Steps 20; Nb bins = 20. We implemented a range of values for the gap width (*X*), between 0.1 and 1.5 to assess the consistency of inferred groups under varying gap width values.

Sequence comparisons with previously known sequences and close species were performed by BLAST (Basic Local Alignment Search Tool; http://www.ncbi.nlm.nih.gov/BLAST) and the BOLD Identification System (IDS) (www.boldsystems.org).

Diagnostic characters among sets of sequences were examined using BOLD’s Diagnostic Character analysis tool. Concordance between BINs assignment and species identification by classical taxonomy was analyzed by the ‘BIN Discordance Report’ sequence analysis tool [5] available on BOLD. The BIN Discordance Report facilitates this check by comparing the taxonomy on selected records against all others in the BINs they are associated with. Specimens corresponding to discordant BINs were re-evaluated by a specialist in order to verify our data and correct potential misidentifications.

## Results

**Fig 2:**
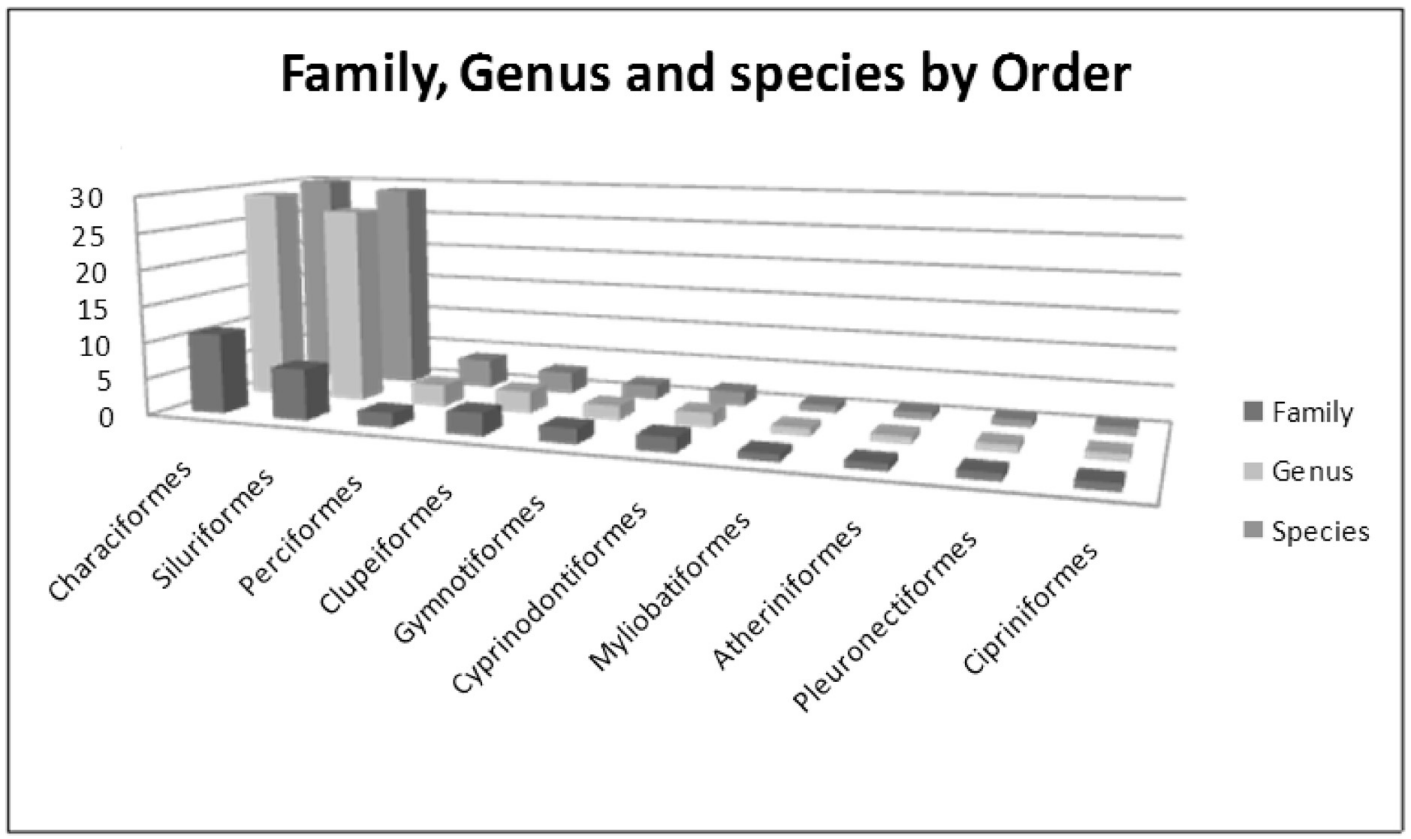
3D histogram showing the number of Families, Genus and species by Order.

Taxonomic identification of the 308 fish specimens resulted in 79 species (71 Genera, 35 Families and 10 Orders) from the Lower Paraná River (Fig 2; S1 Table). All collected species belong to the Class Actinopterygii with the exception of *Potamotrygon motoro* that belongs to the Class Chondrichthyes. Eight out of 71 genera (11.3%) were represented by more than one species (*Astyanax*, *Characidium*, *Odontesthes*, *Pimelodus*, *Ageneiosus*, *Brycon*, *Crenicichla*, and *Cnesterodon*). The number of individuals per species ranged from 1 to 10 (mean 4) with 33 species represented by more than 4 individuals and 16 species represented by one specimen.

Most species of commercial interest were represented in this study, such as the large migratory species *Prochilodus lineatus*, *Salminus brasiliensis*, *Pseudoplatystoma corruscans*, *Brycon orbignyanus* and *Leporinus obtusidens*. Recreational interest species such as *P*. *motoro*, *Hoplias malabaricus*, *Odontesthes bonariensis*, *Gymnotus inaequilabiatus*, *Eigenmannia trilineata*, and *Hoplosternum littorale* were also included. *G*. *inaequilabiatus* and *E*. *trilineata* are widely used as live bait for sport fishing [22]. Most species belong to the orders Siluriformes and Characiformes in agreement with previous reports for Neotropics [58, 59]. In addition, species belonging to the orders Clupeiformes (*Lysengraulis grossidens*, *Ramnogaster melanostoma*, and *Pellona flavipinnis*) and Atheriniformes (*O*. *bonariensis*) that migrate from estuarine or marine environments to freshwater habitats were identified. *Cyprinus carpio*, an exotic species, was also reported.

### DNA barcoding–specimen identification

COI amplified DNA fragments (~ 648 pb) were obtained from all 308 specimens. No stop codons, insertions, or deletions were found in any of the amplified sequences, suggesting that all of them constitute functional mitochondrial COI sequences. No NUMTs (nuclear DNA sequences originating from mitochondrial DNA sequences) amplifications were detected. Average nucleotide frequencies were C (27.53%), T (29.40%), A (24.82%), and G (18.04%), similar to those previously reported in other studies [60].

**Fig 3:**
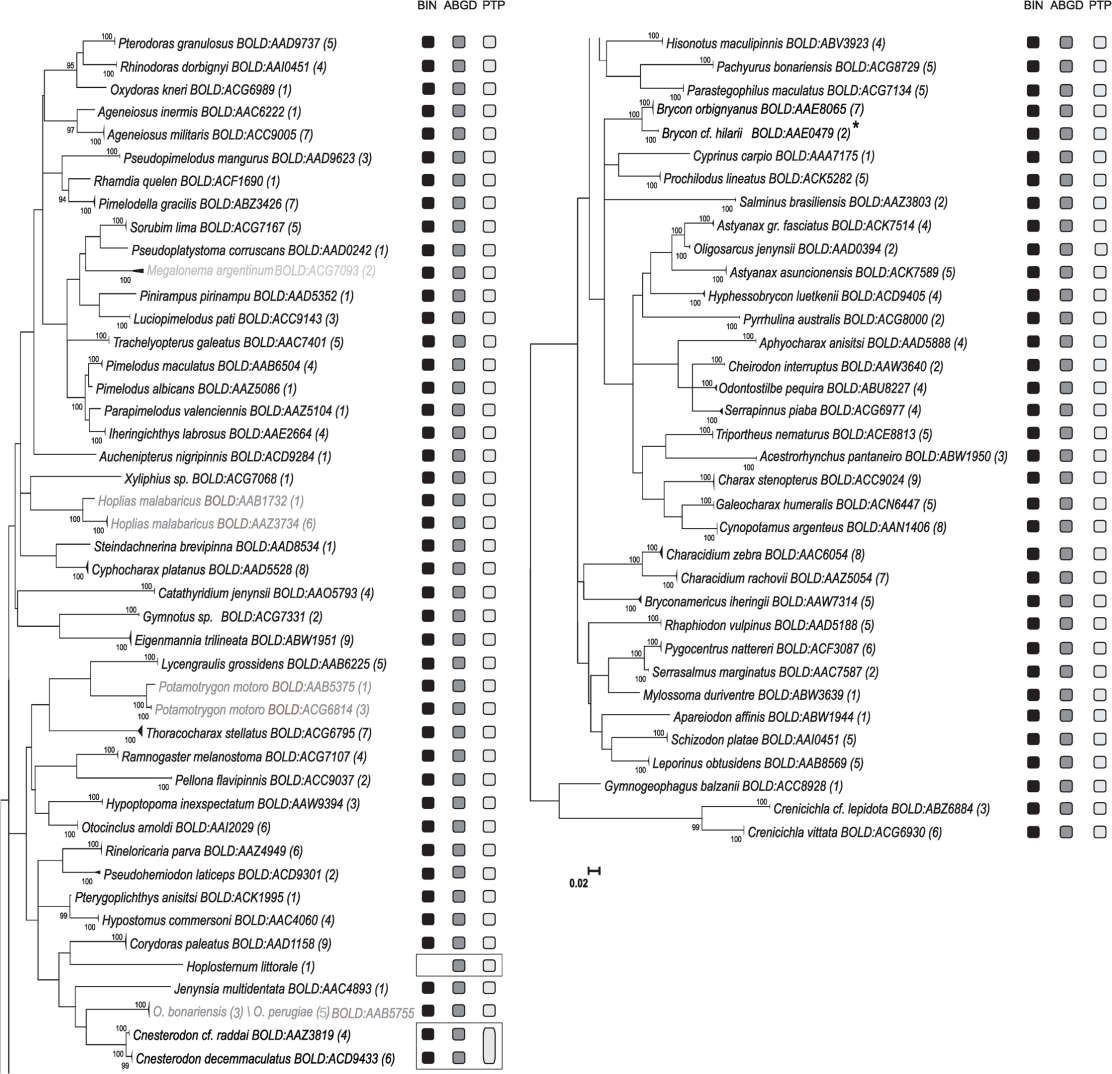
The K2P/ML tree of 308 COI sequences for 79 morphologically identified freshwater fish species from Lower Paraná River in Argentina. NJ tree was divided into two parts from top to bottom in order from left to right. Bootstrap values >90 for 1000 replicates are shown at each branch. The number of specimens analyzed for each species is shown between brackets. Solid triangles represent clusters of multiple specimens, with height proportional to specimen number and the horizontal width proportional to the genetic variation within each cluster. In gray are shown branches of species with high intraspecific genetic divergence and species with overlapping clusters. Columns next to the tree represent presence of recognition for each clustering method while boxes highlights the differences among methods. Specimens of *Brycon orbignyanus* with high genetic divergence that were re-classified as *B*. *cf*. *hilarii* are highlighted with "*".

Relationships among sequences were represented by ML (Fig 3) and NJ (S1 Fig) trees. Both K2P ML and NJ trees grouped sequences of the same taxonomically identified species in no overlapping clusters, with the exception of two species of the genera *Odontesthes* (*O*. *bonariensis* and *O*. *perugiae*) which were present in the same COI cluster. Species clusters were supported with boostrap values of 100%. Deep intraspecific divergences were observed in the NJ and K2P ML analysis among some sequences of *H*. *malabaricus*, *B*. *orbignyanus*, *P*. *motoro* and *Megalonema argentinum* species (Fig 3).

Genetic distances increased from lower to higher taxonomic levels. The average K2P genetic distance between specimens was 0.53% within species, 12.26% within genera and 19.61% within families (Table 1). The average K2P genetic distance within conspecific specimens was 23-fold lower than the average value found in congeneric species.

**Table 1:** K2P genetic divergence values within different taxonomic levels from 308 specimens of Lower Paraná River analyzed.

** **		**K2P genetic divergence (%)**			
** **	**Comparisons**	**Minimun**	**Mean**	**Maximun**	**SE**
**Within Species**	698	0	0.53	7.59	0
**Within Genus**	121	0	12.26	19.98	0.05
**Within Family**	2090	4.3	19.61	28.34	0

SE: standard error

Distances between species ranged from 0.00% to 19.98% (Table 1), considering the two species of the genus *Odontesthes* with a very low genetic distance value ranging from 0% to 0.62% (Table 2). For the other congeneric species, minimum interspecific distances ranged from 4.06% in *Pimelodus* genus to 19.48% in *Crenicichla* genus (Table 2).

**Table 2:** Minimum and maximum genetic distances of Genus with more than one species.

**Genus**	**Number of species**	**Minimum Distance (%)**	**Maximum Distance (%)**
*Odontesthes*	2	0	0.62
*Pimelodus*	2	4.06	4.21
*Ageneiosus*	2	9.78	11.65
*Characidium*	2	12.4	13.73
*Astyanax*	2	15.21	16.77
*Crenicichla*	2	19.48	19.89

**Fig 4:**
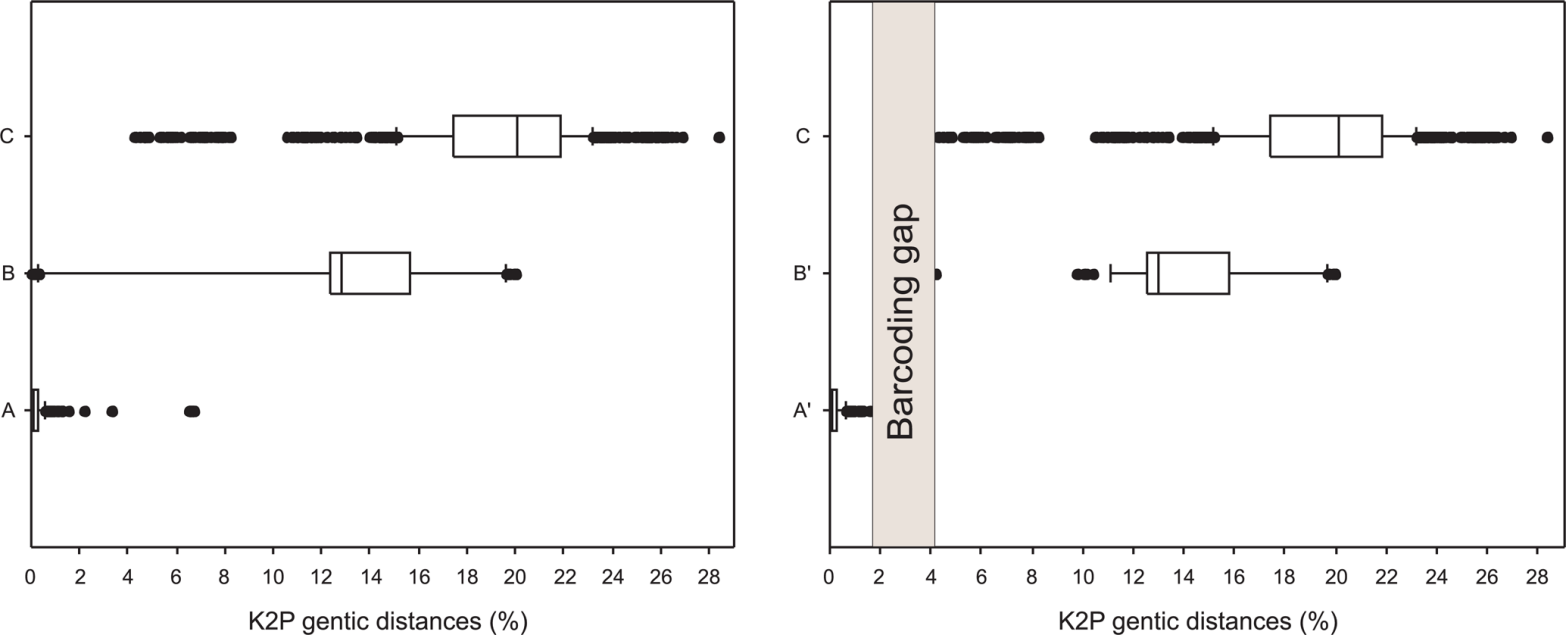
Box plots of K2P distances at different taxonomic levels. (A) within-species variation; (A’) within-species variation excluding the four species with high genetic divergence (*H*. *malabaricus*, *B*. *orbignyanus*, *P*. *motoro* and *Megalonema argentinum*); (B) variation at genus level; (B’) variation at genus level excluding genus with low genetic divergence (*Odontesthes*), (C) variation at Family level. The box comprise 25–75th percentiles of the data set. Whiskers show the lowest and highest values. Points represent outliers. Grey bar indicates ‘barcoding gap’ between intra and interspecific distances.

A barcode gap of 2.5% between conspecifics and congenerics K2P distances was observed for most analyzed data (Fig 4). In 95% of species analysed the maximum intraspecific distance was 1.56%. The remaining 5% presented intraspecific distances higher than 2%: *H*. *malabaricus* (7.59%), *B*. *orbignyanus* (6.68%), *P*. *motoro* (3.32%), and *M*. *argentinum* (2.19%) (Fig 4A and 4A’). The minimum genetic distance between species was 4.06% in 99% of species analysed. Only *Odontesthes* genus presented an intraspecific distance lower than 0.62% (Fig 4B and 4B’).

**Fig 5:**
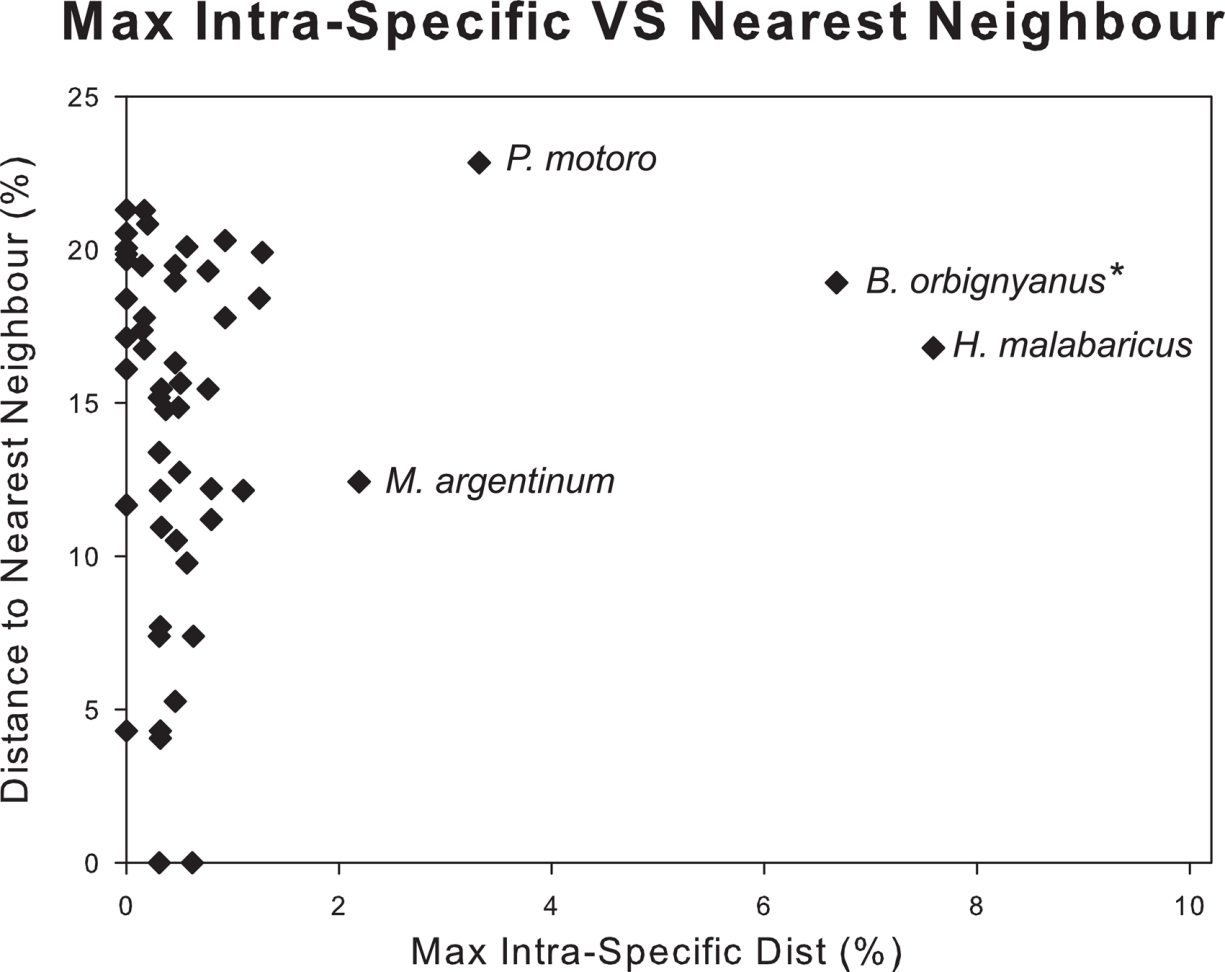
Scatterplot showing the overlap of the max intraspecific distances vs. the interspecific (Nearest Neighbour) distances. Specimens of *Brycon orbignyanus* with high genetic divergence that were re-classified as *B*. *cf*. *hilarii* are highlighted with "*".

The species discrimination power of DNA barcoding was analysed by plotting the maximum intraspecific distance of each species against its minimum distance to the nearest neighbour (Fig 5). For whole data set, with the exception of *Odontesthes* species sequences, genetic distances of each species to their nearest neighbours were higher than the maximum intraspecific genetic distance, showing that COI barcode could discriminate 97% of species analysed from Lower Paraná River (Fig 5).

### Species delimitation

For reliable COI species assignment, all data set was analyzed by three clustering methods (BIN, ABGD and PTP) in order to confirm the concordance between sequence clusters and species designations through taxonomy.

Congruent results were obtained among the three clustering methods tested in most cases. Records were assigned to 79 BINs corresponding to 79 species identified by taxonomy experts (Fig 3). One species (*Hoplosternum littorale)* had no BIN, since it did not meet BOLD minimum requirements to be included in this analysis. Two species shared one same BIN (*O*. *bonariensis* and *O*. *perugiae*). New BINs for *G*. *inaequilabiatus*, *P*. *motoro*, *Crenicichla vittata*, *Serrapinus piaba*, *M*. *argentinum*, and *Pachyurus bonariensis* were generated. *G*. *inaequilabiatus* and *P*. *motoro* have already had records with a different BIN number. The description of new BINs for species that already had records with a different BIN number could reveal possible cryptic fish species or misidentification.

Differences between clustering methods were found in *C*. *decemmaculatus*—*C*. *cf*. *raddai* cluster, in which BIN and ABGD reported two groups while PTP reported only one (Fig 3).

At least two clustering methods separated three of the four taxonomic identified species with higher intraspecific divergence into two different clusters, suggesting that these three groups deserved further investigation (*H*. *malabaricus*, B. *orbignyanus*, and *P*. *motoro*). In the case of *M*. *argentinum*, only one cluster was defined by the three clustering methods. Little information is available about *M*. *argentinum* biology, geographical distribution and conservation status, and no molecular data has been reported until now.

### Special cases

#### Two clusters for one taxonomic identified species

**Fig 6:**
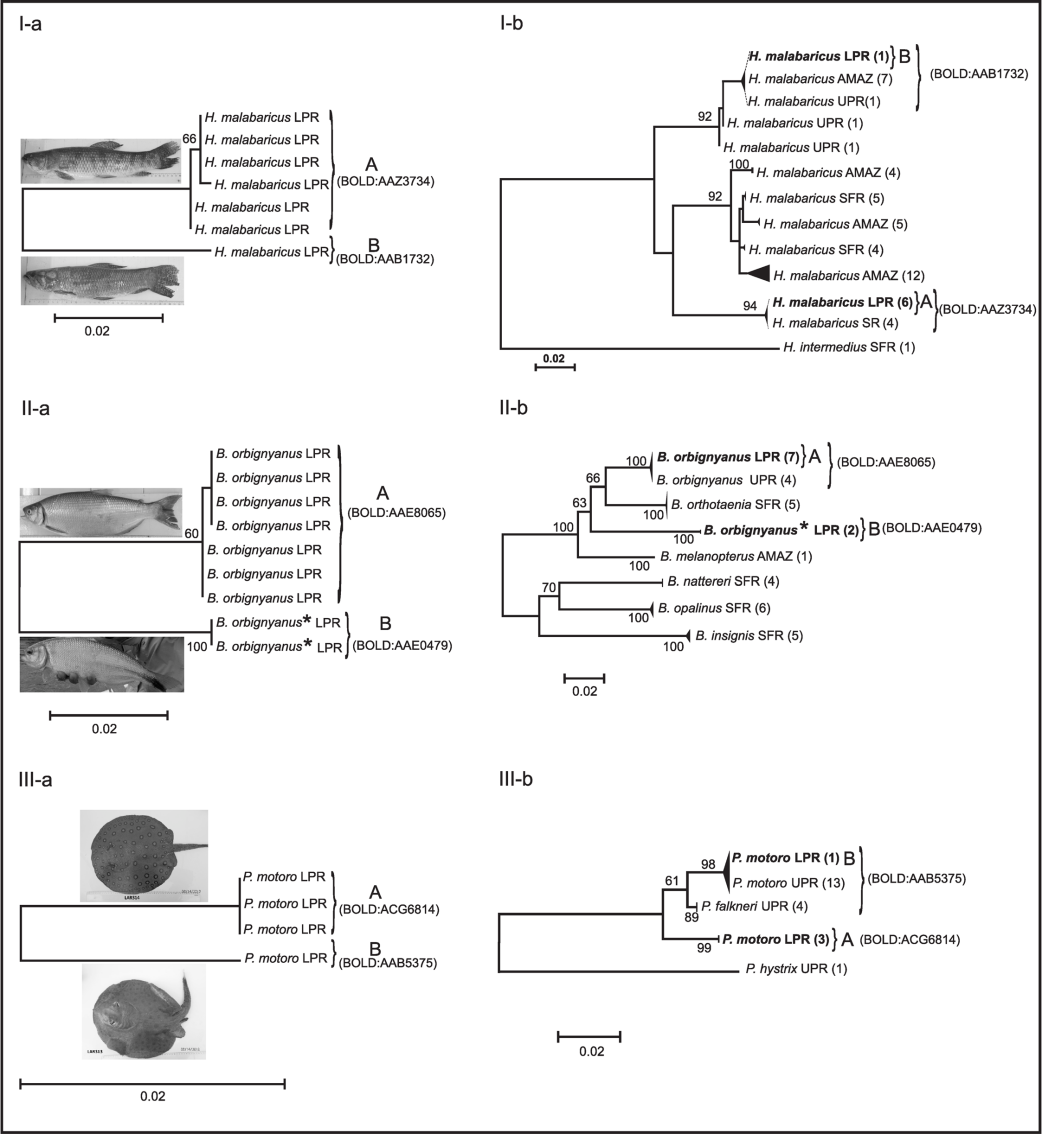
K2P- NJ trees of species with high conspecific genetic divergence. On the left, K2P- NJ trees calculated with specimens of this project. On the right, K2P-NJ trees calculated with all the South American published sequences of each genus. Specimens of this project are shown in bold and clusters indicated with A and B. BIN numbers are shown in brackets. Pictures represent morphology of typical specimens for each branch. Specimen number on collapsed branches is shown in brackets. Bootstrap values >50 for 1000 replicates are shown at each branch. Specimens of *Brycon orbignyanus* with high genetic divergence that were re-classified as *B*. *cf*. *hilarii* are highlighted with "*". LPR: Lower Paraná River, UPR: Upper Paraná River, AMAZ: Amazonas River, SR: Salado River and SFR: Sao Francisco River.

For further analyses of *H*. *malabaricus*, *B*. *orbignyanus*, and *P*. *motoro* cases, K2P-NJ trees were performed (Fig 6) using COI sequences obtained in this work and published sequences (published papers or public BOLD projects) of the same genus.

*The Hoplias case*. *H*. *malabaricus* sequences (n = 54) from four different South American river basins were retrieved, namely Salado River (SR, BOLD project code FIPP), Sao Francisco River (SFR, BOLD project code BSB), Upper Paraná River (UPR, BOLD project code FUPR) and Amazonas River (AMAZ, [61]). NJ tree was rooted using an *H*. *intermedius* sequence from BSB-BOLD project. Results showed that the six sequences that grouped together in the general tree (Fig 3 and Fig 6I-a cluster A) clustered with sequences of *H*. *malabaricus* from Salado River basin (Fig 6I-b). This cluster presented a maximum divergence of 0.3% and conformed only one BIN (BOLD:AAZ3734). The sequence in a separated branch at the main tree (Fig 3 and Fig 6I-a cluster B), clustered with *H*. *malabaricus* sequences from Upper Parana River and Amazonas River basins with a maximum divergence of 0.5%, conforming another BIN (BOLD:AAB1732) (Fig 6I-b). In addition, this sequence belonged to a specimen with different phenotypic characteristics easily recognized by fishermen (*H*. *malabaricus* of “small head", Fig 6I-a, photographs). For sampling areas covered in this work only *H*. *malabaricus* has been cited. Our results showed that deep genetic divergence for *H*. *malabaricus* also occurred in a smaller geographic scale since all individuals were sampled in the same area. Genetic and/or morphological-morphometric studies have not been reported yet for *H*. *malabaricus* from Lower Paraná River. Therefore, information obtained in this work would provide the kickoff for future research on this species complex.

*The Brycon genus case*. Only published sequences (n = 33) of six South American species were used: *B*. *orbignyanus* from Upper Paraná River (BOLD project code FUPR), *Brycon melanopterus* from Amazonas River and *Brycon orthotaenia*, *Brycon opalinus*, *Brycon insignis and Brycon nattereri* from Sao Francisco River (BOLD project code BSB). Comparative NJ sub-tree showed that seven COI sequences of *B*. *orbignyanus* specimens that grouped together in the general tree (Fig 3 and Fig 6II-a-cluster A) clustered with *B*. *orbignyanus* sequences from Upper Paraná River (Fig 6II-b). This group presented a mean K2P distance of 0.07% and a maximum distance of 0.16%, and belonged to the same BIN (BOLD:AAE8065). Interestingly, the other two specimens that conformed cluster B at the NJ tree in Fig 6II-a, conformed a cohesive separate cluster with a different BIN number (BOLD:AAE0479) (Fig 6II-b). In order to determine species-specific status, both sequences were compared by BLAST against the NCBI nr database and by IDS tool against BOLD database. Both sequences displayed a 99% identity with *B*. *hilarii* sequences and over 99.6% of similarity by IDS tool, showing that the vouchers might be misidentified. Only photographic vouchers were available for these two individuals, which were revised and compared with all *B*. *orbignyanus* photographic vouchers. Taking into account sequence comparison results and differences found between photographic vouchers, both BOLD records were re classified as *Brycon cf*. *hilarii* in BOLD database and in NJ tree (Fig 3). Although *B*. *hilarii* distribution corresponds to Paraguay River Basin [62], our result showed that *B*. *hilarii* could reach the Paraná River near the mouth of the Paraguay River (Fig 1, sampling site 2). However, a higher sampling effort is necessary to confirm this observation.

*The P*. *motoro case*. Two clusters were observed at the main tree with deep intraspecific divergences between them (Fig 3 and Fig 6III-a). One cluster (A) grouped three sequences (BIN BOLD:ACG6814) while the other one (B), included only one sequence (BIN BOLD:AAB5375). Comparison analysis of COI sequences was performed using sequences of three different species from Upper Paraná River (*P*. *motoro* and *Potamotrygon falkneri* of BOLD project code FUPR, and *Potamotrygon hystri*x [63]). Comparison analysis (Fig 6III-b) revealed that the sequence at cluster B grouped with *P*. *motoro and P*. *falkneri* sequences from the Upper Paraná River conforming a single BIN (BOLD:AAB5375). The other three *P*. *motoro* sequences (cluster A) were displayed in a separated branch, without genetic divergence among them, and constituted a new exclusive BIN (BOLD:ACG6814). Interestingly, the three *P*. *motoro* sequences in cluster A did not cluster to any other known species of the genus with reported COI sequences. A possible explanation for this result could be the presence of a new species that share some morphological characters and pattern coloration with *P*. *motoro*, which has led to misidentification. Vouchers revision was not possible in this case since only photographic vouchers were available for these individuals.

#### More than one species for one cluster: Species with interspecific genetic divergence values in the same range than intraspecific genetic divergence values: *O*. *perugiae* and *O*. *bonariensis*

COI interspecific genetic K2P distance between *O*. *perugiae* and *O*. *bonariensis* was in the same range as intraspecific distances recorded for *O*. *bonariensis*. BOLD’s Diagnostic Character analysis showed that there was not any exclusive nucleotide for species. Six haplotypes were observed among *O*. *perugiae* and *O*. *bonariensis* sequences, two of them were shared between specimens from both species (data not shown). Moreover, COI haplotypes were shared also with other *Odontesthes* species, such as *O*. *argentinensis* and *O*. *mirinensis* (Villanova GV not published results).

#### Barcode Index Numbers (BINs) and taxonomic concordance

To check the correlation degree between species designations by taxonomy and assigned BINs, all sequences were analyzed by the 'BIN Discordance Report' tool available within the "Sequence Analysis" module of BOLD. As a result 79 BINS were identified from 291 records that met the minimum requirements to be included in the analysis. Taxonomic concordance was found in 113 records of 27 BINs (34.2%), 1 record (*Xyliphius sp*.) was singleton (BINs with single specimens) (1.2%) and 177 records of 51 BINs (64.6%) were conflictive, indicating that at least two different taxonomic assignments were found in BOLD database within a single BIN.

Among the 51 discordant BINS, 6 were at the Family level (17 conflicting records), 16 at the Genus level (60 conflicting records) and 29 at the species level (100 conflicting records). After an exhaustive revision by two independent groups of fish taxonomists, who worked with identification reliability level 2 according to the Fish-BOL collaborator’s protocol [28], the 17 conflicting records at the Family level as well as the 60 records at the Genus level were resolved. In most of these cases, the discordant entries were caused by misidentifications in previous BOLD data projects. Out of 29 discordant BINS at the species level, 15 were shown to exhibit ‘no true’ discordances, as the discordant BINS were caused by lack of taxonomic determination at species level in previous BOLD projects (S2 Table) such as species identified as sp. or cf. (e.g. *Potamotrygon sp*., *Pimelodella cf*. *cristata*, etc.).

There were 28 conflicting taxa within the 14 “true” discordant BINS. Among these conflicting taxa common characteristics were found and records were classified in four groups in order to explain the discordance among records within a BIN (Table 3): Group I (*): *the discordance was probably caused by misidentifications*,; Group II (**): *the discordance was probably caused by COI lower species-specific power of discrimination*; Group III (***): *the discordance could not be analyzed comparing involved records since COI sequences were not available in public BOLD projects*. BIN discordance analysis in BOLD is performed using all record uploaded to BOLD database. However a high proportion of these records belonged to projects that are not public and sequences were not available to be used. Group IV (****): *the discordance was probably caused by a combination of tree previous cases*. For each conflicting sequence data, the maximum intraspecific distance and nearest neighbour distance were calculated and geographic distribution of specimens and references were added when available (Table 3).

**Table 3:** List of species with “true” discordance BINs found by the ‘BIN Discordance Report’ sequence analysis tool. Only BINs with species level conflicts are shown.

**Identification**	**Conflicting taxon in BIN**	**BIN**	**Country**	MI and NN Distances^#^	**Conflicting Taxon distribution in LPR**	**References**
Ageneiosus inermis*	*Ageneiosus ucayalensis*	BOLD:AAC6222	Brazil	MID: 2.41%—NND: 6.86%	No	[64]
Leporinus obtusidens*	*Leporinus piavussu*	BOLD:AAB8569	Brazil	MID: 4.17%—NND: 2.67%	No	[65]
Eingenmannia trilineata*	*Eigenmania virescens*	BOLD:ABW1951	Argentina	MID: 1.11%—NND: 2.41%	Yes	[10]
Pseudoplatystoma corruscans*	*Pseuplatystoma reticulatum*	BOLD:AAD0242	N/D	MID: 1.71%—NND: 4.33%	Yes	[10]
Serrasalmus marginatus****	*Serrasalmus rhombeus*	BOLD:AAC7587	Brazil—Guyana	MID: 1.96—NND: 1.96	No	[66]
*** ***	*Serrasalmus eingenmanni*	BOLD:AAC7587	Bolivia	MID: 1.96—NND: 1.96	No	[66]
*** ***	*Serrasalmus hollandi*	BOLD:AAC7587	Bolivia	MID: 1.96—NND: 1.96	No	NR
*** ***	*Serrasalmus compressus*	BOLD:AAC7587	Bolivia	MID: 1.96—NND: 1.96	No	NR
Odontesthes bonariensis****	*Odontesthes perugiae*	BOLD:AAB5755	Argentina	MID: 2.09%—NND: 2.41%	Yes	[67]
*** ***	*Odontesthes argentinensis*	BOLD:AAB5755	Argentina—Uruguay—Brazil	MID: 2.09%—NND: 2.41%	No	[67]
*** ***	*Odontesthes humensis*	BOLD:AAB5755	Argentina—Uruguay—Brazil	MID: 2.09%—NND: 2.41%	No	[67]
*** ***	*Odontesthes mauleanum*	BOLD:AAB5755	Chile	MID: 2.09%—NND: 2.41%	No	NR
*** ***	*Odontesthes platensis*	BOLD:AAB5755	Argentina	MID: 2.09%—NND: 2.41%	No	[67]
*** ***	*Odontesthes hatcheri*	BOLD:AAB5755	Argentina	MID: 2.09%—NND: 2.41%	No	[67]
Prochilodus lineatus****	*Prochilodus nigricans*	BOLD:AAB5650	Brazil-Bolivia	MID: 3.68%—NND: 7.25%	No	This work (S2 Fig)
*** ***	*Prochilodus costatus*	BOLD:AAB5650	Brazil	MID: 3.68%—NND: 7.25%	No	[68]
*** ***	*Prochilodus argenteus*	BOLD:AAB5650	Brazil	MID: 3.68%—NND: 7.25%	No	[68]
*** ***	*Prochilodus rubrotaeniatus*	BOLD:AAB5650	Guyana	MID: 3.68%—NND: 7.25%	No	NR
*** ***	*Prochilodus hartii*	BOLD:AAB5650	Brazil	MID: 3.68%—NND: 7.25%	No	NR
Potamotrygon motoro**	*Potamotrygon falkneri*	BOLD:AAB5375	Brazil—Peru—Argentina	MID: 2.91%—NND: 1.69%	Yes	[69]
Acestrorhynchus pantaneiro***	*Acestrorhynchus altus*	BOLD:ABW1950	Brazil	MID: 0.5%—NND: 3.35%	No	NR
Auchenipterus nigripinnis***	*Auchenipterus brachyurus*	BOLD:ACD9284	Bolivia	MID: 0.77%—NND: 2.73%	No	NR
Triportheus nematurus***	*Triportheus pantanensis*	BOLD:ACE8813	Brazil	MID: 1.13%—NND: 1.61%	Yes	NR
Schizodon platae***	*Schizodon jacuiensis*	BOLD:ACG9260	Brazil	MID: 0%- NND: 6.42%	No	NR
Rineloricaria parva***	*Rineloricaria aurata*	BOLD:AAZ4949	Brazil	MID: 0.94%—NND: 6.21%	No	NR
*** ***	*Rineloricaria lima*	BOLD:AAZ4949	Argentina	MID: 0.94%—NND: 6.21%	Yes	NR
Pimelodella gracilis***	*Pimelodella laticeps*	BOLD:ABZ3426	Argentina	MID: 0.92%—NND: 1.84%	Yes	NR
*** ***	*Pimelodella taenioptera*	BOLD:ABZ3426	Argentina	MID: 0.92%—NND: 1.84%	Yes	NR

MID: Maximum Intraspecific Distance; NND: Nearest Neighbour Distance; N/D: No data; NR: No register

^#^: values obtained using individuals grouped in the same BIN

*: *the discordance was probably caused by misidentifications*

**: *the discordance was probably caused by COI lower* species-specific power of discrimination

***: the discordance COI sequences were not published or available in public BOLD projects

****: the discordance was probably caused by a combination of tree previous cases.

## Discussion

### Barcoding success

The present study represents the first molecular survey of Lower Paraná River fish diversity corresponding to the Southernmost Neotropical region. Seventy nine (43%) of the 185 fish species described for the Lower Paraná River were assessed in this work using COI barcodes from a sub-area of this river. The observed COI genetic distances between conspecifics and congenerics (means: 0.53% and 12.26% respectively) for Lower Paraná River fish were within the range of previously reported values from fishes of freshwater ecosystems [8, 10, 27, 70]. All sequences of the same species formed high bootstrap-supported clusters without any overlap between species, even in species within the same genera, with the exception of *Odontesthes* species. Nine new records not previously studied by COI barcodes were generated and incorporated to the BOLD data system (*C*. *cf*. *raddai*, Schizodon platae*, *Otocinclus arnoldi*, Parastegophilus maculatus*, *Pseudohemiodon laticeps*, *M*. argentinum*, *Auchenipterus nigripinnis*, *B*. *cf*. *hilarii and Xyliphius sp*.), three of which are endemic species of Argentina (*). Our study included species that migrate from estuarine or marine environments to freshwater habitats. Reproductive activity was reported for some of them in the Lower Paraná River (e.g. *L*. *grossidens* [71]). In this regards, COI barcode reference library will contribute to future freshwater ichthyoplankton identification and life cycle monitoring.

### Species delimitation and data reliability

Molecular data provide a valuable resource for preliminary species delimitations or validating traditional phenotype-based species circumscriptions [7, 72]. In our study, all data set was analyzed by three clustering methods and numerous experts in order to confirm the concordance between sequence clusters and species designations by taxonomy. Analysis of the results obtained by distinct clustering methods offer an additional level of confidence in the inferred OTUs in Lower Paraná River fish. Two species (*H*. *malabaricus* and *P*. *motoro*) displayed a deep intra-specific genetic distance (>2%) and the corresponding sequences grouped into two different clusters in each one.

The genus *Hoplias* is distributed throughout many hydrographic systems of South America and has 11 recognized species. At least 3 of them are present in Argentina [73, 74]. Only *H*. *malabaricus* was described in the Lower Parana River. *H*. *malabaricus* is considered by many authors as a complex of cryptic species that require a profound taxonomic revision [61, 75]. Based on COI sequence analysis, a strong geographic structure for *H*. *malabaricus* from distant hydrographic basins in South America was previously proposed [27]. However, specimens obtained in this study from the same Paraná River area also present high COI divergence supporting the hypothesis of the existence of a cryptic species in the Lower Paraná River.

At least six freshwater stingrays species of the genera *Potamotrygon* are present in the Lower Parana River (*P*. *schuhmacheri*, *P*. *hystrix*, *P amandae*, *P*. *brachyura*, *P*. *falknerii*, and *P*. *motoro*). Recently, a new *Potamotrygon sp*. was described by Almirón *et al*. [18], in the Paraná Delta area, which could not be assigned to previously described species. Among them, *P*. *motoro* is the most widely distributed species of the family Potamotrygonidae, present in most freshwater systems in South America [63, 76, 77]. The widespread distribution of the genera *Potamotrygon*, together with significant variation in some morphological characters (*e*.*g*., dorsal disc coloration) among populations of different basins, and even in closely adjacent areas, has led some authors to indicate that a taxonomic subdivision of *P*. *motoro* may be necessary [77, 78]. In the case of Lower Paraná River stingrays, only COI sequences for *P*. *motoro* are available. Although low intra and interspecific variation have been reported among Potamotrygonidae family members in the Upper Paraná River basin [10, 79, 80], our results showed a high COI divergence between *P*. *motoro* specimens. This scenario claims a more extensive and profound taxonomic revision along with DNA sequences analysis for *Potamotrygon* genera that inhabits the Lower Parana River.

Results obtained in this work, and previous reports [10, 11, 22] state the existence of hidden diversity in many species and suggest that Neotropical species richness is still underestimated.

### Importance of reference libraries

The success of using barcoding for species identification strongly depends on the presence of high-quality reference sequences available in public sequence libraries and the existence of specimen vouchers correctly identified. DNA barcode databases such as BOLD, have implemented minimal quality criteria for barcode data acquisition and generation [24]. However, reference specimen misidentification appears to be the single largest factor contributing to errors in the FISH-BOL data set [81]. Barcoding methodology and a careful examination of specimens allowed us to resolve apparent outliers and cluster conflicts in the FISH-BOL data set. This situation also highlights the importance of checking the taxonomic identity in the light of COI information previous to its upload in public sequence databases. This was the case for *Brycon cf*. *hilarii*, previously characterized as *B*. *Orbignyanus*, as well as for several detected BIN discordances based in misidentification. In these cases, the analyses of COI sequences correctly separates species pairs obtained in previous barcode studies on these species (e.g. *Ageneiosus inermis vs*. *Ageneiosus ucayalensis* [64]).

COI sequence comparisons was not able to discriminate between two species of the genera *Odontesthes* (*O*. *bonariensis* and *O*. *perugie*). Moreover, shared haplotypes between specimens of both species were found (data not shown). This result is consistent with a recent radiation process in the genera *Odontesthes* as proposed by Garcia *et al*. (2014) [67] and Campanella *et al*. (2015) [82], and states that COI information could be used only for genera identification.

The current COI reference library provides highly reliable DNA and vouchers exemplars for 97.5% of the fish species investigated and it can be confidently used as a benchmark for identification of almost 50% of Lower Parana River fish species. This COI barcode library will be especially important for fish biodiversity monitoring, for sustainable exploitation of fishing resources, for reproductive biology studies and ecological monitoring, among other applications.

## Supporting Information

S1 Fig The K2P/NJ tree of 308 COI sequences for 79 morphologically identified freshwater fish species from the Lower Paraná River in Argentina. Bootstrap values for 1000 replicates are shown at each branch. Before and after of the species name voucher and BIN numbers are respectively shown. Specimens of *Brycon orbignyanus* with high genetic divergence that were re-classified as *B*. *cf*. *hilarii* are highlighted with "*".
(TIF)

S2 Fig *Prochilodus* genus K2P/NJ tree showing that COI clearly separates *P*. *lineatus* and *P*. *nigricans* species. Bootstrap values for 1000 replicates are shown at each branch. The number of specimens analyzed for each species is shown between brackets. Solid triangles represent clusters of multiple specimens, with height proportional to specimen number and the horizontal depth proportional to the genetic variation within each cluster.
(TIF)

S1 Table Taxonomic classification of the 79 morphologically identified freshwater fish species from the Lower Paraná River in Argentina. (DOCX)

S2 Table List of species with “no true” discordance BINs found by the ‘BIN Discordance Report’ sequence analysis tool. Only BINs with species level conflicts are shown.
(DOCX)
