# Supplementary figures and images for: First DNA Barcode Reference Library for the Identification of South American Freshwater Fish from the Lower Paraná River
Source: PLoS One. 2016 Jul 21;11(7):e0157419. doi: 10.1371/journal.pone.0157419 (PMC4956254; doi:10.1371/journal.pone.0157419)

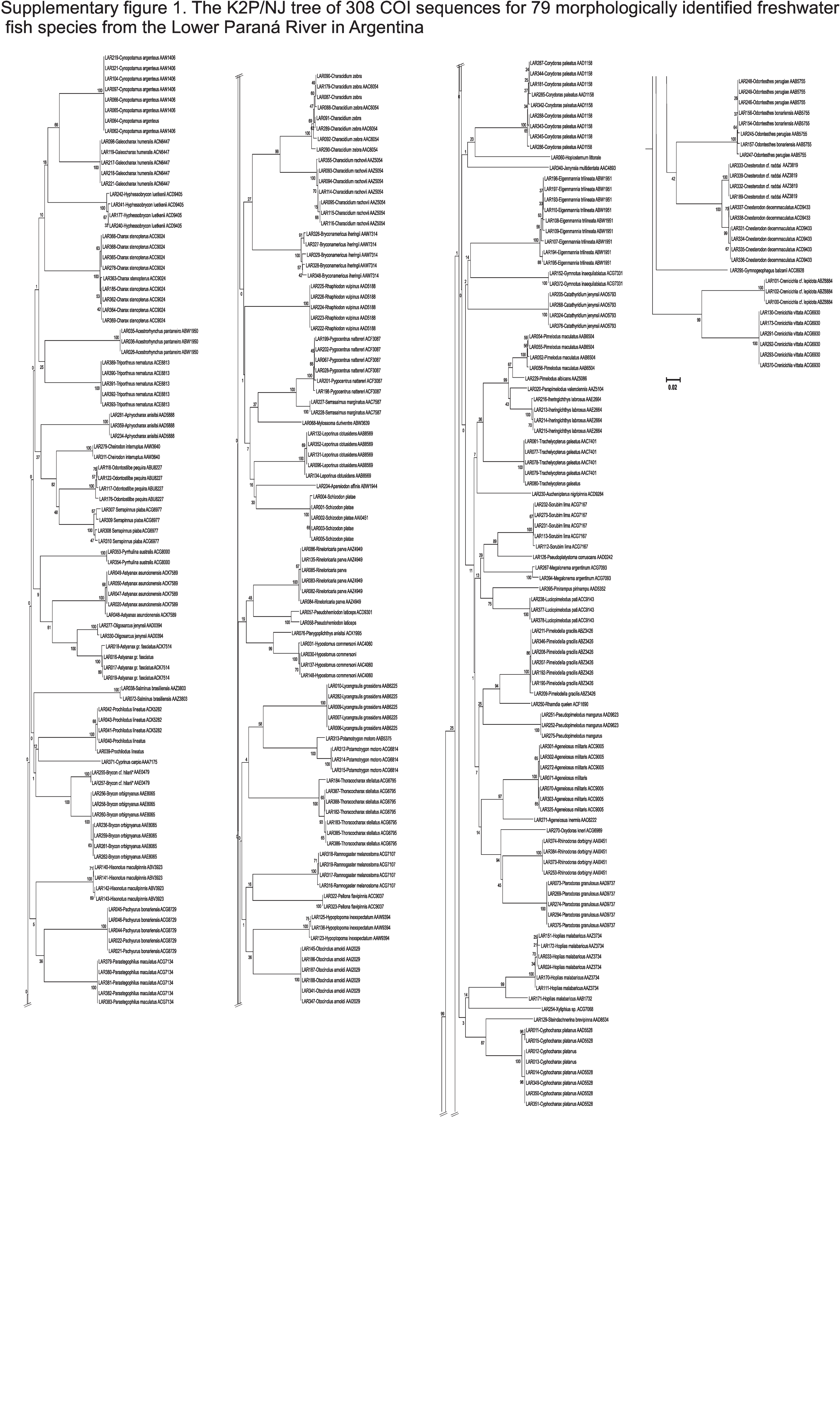

Supplement: S1 Fig — Bootstrap values for 1000 replicates are shown at each branch. Before and after of the species name voucher and BIN numbers are respectively shown. Specimens of Brycon orbignyanus with high genetic divergence that were re-classified as B. cf. hilarii are highlighted with "*". (TIF) [file pone.0157419.s001.tif]

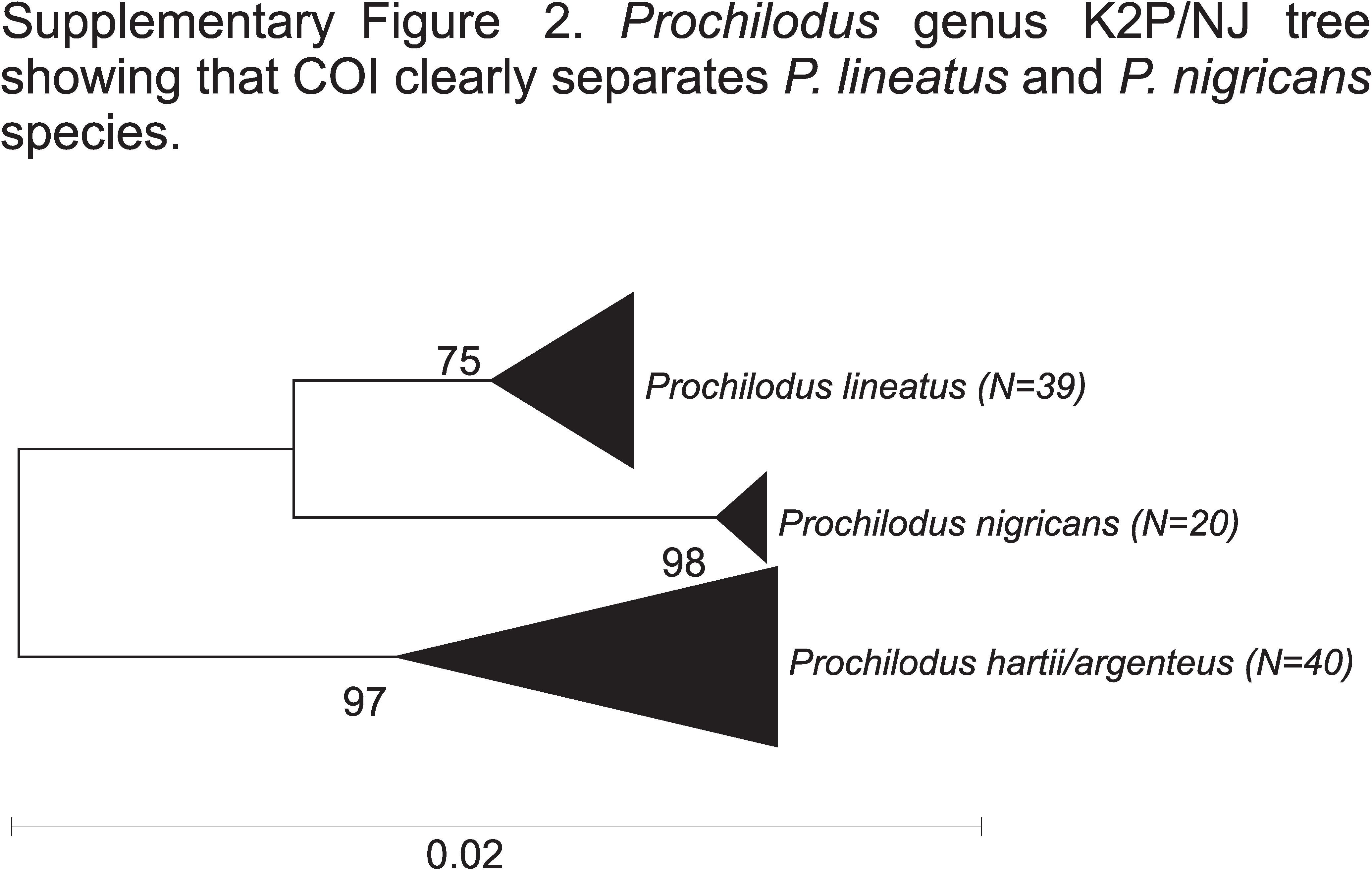

Supplement: S2 Fig — Bootstrap values for 1000 replicates are shown at each branch. The number of specimens analyzed for each species is shown between brackets. Solid triangles represent clusters of multiple specimens, with height proportional to specimen number and the horizontal depth proportional to the genetic variation within each cluster. (TIF) [file pone.0157419.s002.tif]
