# Supplementary material for: First DNA Barcode Reference Library for the Identification of South American Freshwater Fish from the Lower Paraná River
Source: PLoS One. 2016 Jul 21;11(7):e0157419. doi: 10.1371/journal.pone.0157419 (PMC4956254; doi:10.1371/journal.pone.0157419)
Supplement: S1 Table — (DOCX) [file pone.0157419.s003.docx]

Supplementary Table 1: Taxonomic classification of the 79 morphologically identified freshwater fish species from the Lower Paraná River in Argentina.

| Class | Order | Family | Species |
| --- | --- | --- | --- |
| Chondrichthyes | Myliobatiformes | Potamotrygonidae | *Potamotrygon motoro* |
| Osteichthyes | Clupeiformes | Clupeidae | *Ramnogaster melanostoma* |
|  |  | Engraulididae | *Lycengraulis grossidens* |
|  |  | Pristigasteridae | *Pellona flavipinnis* |
|  | Characiformes | Parodontidae | *Apareiodon affinis* |
|  |  | Curimatidae | *Steindachnerina brevipinna* |
|  |  |  | *Cyphocharax platanus* |
|  |  | Prochilodontidae | *Prochilodus lineatus* |
|  |  | Anostomidae | *Schizodon platae* |
|  |  |  | *Leporinus obtusidens* |
|  |  | Crenuchidae | *Characidium rachovii* |
|  |  |  | *Characidium zebra* |
|  |  | Gasteropelecidae | *Thoracocharax stellatus* |
|  |  | Characidae | *Astyanax gr.fasciatus* |
|  |  |  | *Astyanax asuncionensis* |
|  |  |  | *Bryconamericus iheringii* |
|  |  |  | *Hypressobrycon luetkeni* |
|  |  |  | *Oligosarcus jenynsii* |
|  |  |  | *Salminus brasiliensis* |
|  |  |  | *Triportheus nematurus* |
|  |  |  | *Serrasalmus marginatus* |
|  |  |  | *Pygocentrus nattereri* |
|  |  |  | *Mylossoma durivente* |
|  |  |  | *Aphyocharax anisitsi* |
|  |  |  | *Charax stenopterus* |
|  |  |  | *Cynopotamus argenteus* |
|  |  |  | *Galeocharax humeralis* |
|  |  |  | *Cheirodon interruptus* |
|  |  |  | *Serrapinus piaba* |
|  |  |  | *Odontostilbe pequira* |
|  |  |  | *Brycon orbignyanus* |
|  |  |  | *Brycon cf. hilarii* |
|  |  | Acestrorhynchidae | *Acestrorhynchus pantaneiro* |
|  |  | Cynodontidae | *Rhaphiodon vulpinus* |
|  |  | Erythrinidae | *Hoplias malabaricus* |
|  |  | Lebiasinidae | *Pyrrhulina australis* |
|  | Siluriformes | Aspredinidae | *Xyliphius sp.* |
|  |  | Trichomycteridae | *Parastegophilus maculatus* |
|  |  | Callichthyidae | *Corydoras paleatus* |
|  |  |  | *Hoplosternum littorale* |
|  |  | Loricariidae | *Hypoptopoma inexpectatum* |
|  |  |  | *Hisonotus maculipinnis* |
|  |  |  | *Otocinclus arnoldi* |
|  |  |  | *Rineloricaria parva* |
|  |  |  | *Pseudohemiodon laticeps* |
|  |  |  | *Hypostomus commersoni* |
|  |  |  | *Pterygoplicthys anisitsi* |
|  |  | Pimelodidae | *Iheringichthys labrosus* |
|  |  |  | *Parapimelodus valenciennis* |
|  |  |  | *Pimelodella gracilis* |
|  |  |  | *Pimelodus albicans* |
|  |  |  | *Pimelodus maculatus* |
|  |  |  | *Pinirampus pirinampu* |
|  |  |  | *Luciopimelodus pati* |
|  |  |  | *Megalonema argentinum* |
|  |  |  | *Pseudoplatystoma corruscans* |
|  |  |  | *Sorubim lima* |
|  |  |  | *Rhamdia quelen* |
|  |  | Pseudopimelodidae | *Pseudopimelodus mangurus* |
|  |  | Doradidae | *Pterodoras granulosus* |
|  |  |  | *Oxydoras kneri* |
|  |  |  | *Rhinodoras dorbignyi* |
|  |  | Auchenipteridae | *Ageneiosus inermis* |
|  |  |  | *Ageneiosus militaris* |
|  |  |  | *Auchenipterus nigripinnis* |
|  |  |  | *Trachelyopterus galeatus* |
|  | Gymnotiformes | Gymnotidae | *Gymnotus inaequilabiatus* |
|  |  | Sternopygidae | *Eigenmannia trilineata* |
|  | Atheriniformes | Atherinopsidae | *Odontesthes perugiae* |
|  |  |  | *Odontesthes bonariensis* |
|  | Cyprinodontiformes | Anablepidae | *Jenynsia multidentata* |
|  |  | Poecilidae | *Cnesterodon decemmaculatus* |
|  |  |  | *Cnesterodon cf. raddai* |
|  | Perciformes | Cichlidae | *Crenicichla vittata* |
|  |  |  | *Crenicichla cf. lepidota* |
|  |  |  | *Gymnogeophagus balzanii* |
|  |  | Sciaenidae | *Pachyurus bonariensis* |
|  | Pleuronectiformes | Achiridae | *Catathyridium jenynsii* |
|  | Cypriniformes | Cyprinidae | *Cyprinus carpio* |
