# Supplementary material for: First DNA Barcode Reference Library for the Identification of South American Freshwater Fish from the Lower Paraná River
Source: PLoS One. 2016 Jul 21;11(7):e0157419. doi: 10.1371/journal.pone.0157419 (PMC4956254; doi:10.1371/journal.pone.0157419)
Supplement: S2 Table — Only BINs with species level conflicts are shown. (DOCX) [file pone.0157419.s004.docx]

Supplementary Table 2. List of species with “no true” discordance BINs found by the ‘BIN Discordance Report’ sequence analysis tool. Only BINs with species level conflicts are shown.

| Identification | Conflicting taxon in BIN | BIN | Country | MI and NN Distances* |
| --- | --- | --- | --- | --- |
| *Acestrorhynchus pantaneiro* | *Acestrorhynchus sp.* | BOLD:ABW1950 | Brazil - Argentina - Peru | MID: 0.5% - NND: 3.35% |
| *Auchenipterus nigripinnis* | *Auchenipterus sp.* | BOLD:ACD9284 | Argentina | MID: 0.77% - NND: 2.73% |
| *Characidium zebra* | *Characidium sp.* | BOLD:AAC6054 | Argentina | MID: 2.89% - NND: 3.85% |
| *Cheirodon interruptus* | *Cheirodon sp.* | BOLD:AAW3640 | Argentina | MID: 0.96 - NND: 5.29% |
| *Cnesterodon decemmaculatus* | *Cnesterodon sp.* | BOLD:ACD9433 | Argentina | MID: 0.47% - NND: 2.34% |
| *Cnesterodon cf. raddai* | *Cnesterodon sp.* | BOLD:AAZ3819 | Argentina | MID: 0.46% - NND: 2.41% |
| *Corydoras paleatus* | *Corydoras cf. paleatus* | BOLD:AAD1158 | Argentina | MID: 2.96% - NND: 4.65% |
|  | *Corydoras sp.* |  |  |  |
| *Gymnogeophagus balzanii* | *Gymnogeophagus sp.* | BOLD:ACC8928 | Argentina | MID: 0.8% - NND: 8.1% |
| *Hoplias malabaricus* | *Hoplias sp. vieirai* | BOLD:AAZ3734 | Brazil | MID: 1.82% - NND: 5.54% |
|  | *Hoplias sp.* |  | Argentina |  |
| *Hoplias malabaricus* | *Hoplias sp.* | BOLD:AAB1732 | Argentina | MID: 3.54% - NND: 5.35% |
| *Hypoptopoma inexpectatum* | *Hypoptopoma cf. inexpectatum CAC-2008* | BOLD:AAW9394 | ND | MID: 0.64% - NND: 4.39% |
| *Jenynsia multidentata* | *Jenynsia sp.* | BOLD:AAC4893 | Argentina | MID: 1.84% - NND: 17.01% |
| *Leporinus obtusidens* | *Leporinus cf. obtusidens A GSAS-2007* | BOLD:AAB8569 | Brazil | MID: 4.17% - NND: 2.67% |
|  | *Leporinus cf. obtusidens B GSAS-2007* |  |  |  |
| *Odontesthes bonariensis* | *Odontesthes cf. argentinensis* | BOLD:AAB5755 | Argentina | MID: 2.09% - NND: 2.41 |
|  | *Odontesthes cf. bonariensis* |  |  |  |
|  | *Odontesthes sp.* |  |  |  |
|  | *Odontesthes cf. perugiae* |  |  |  |
| *Pimelodella gracilis* | *Pimelodella cf. cristata* | BOLD:ABZ3426 | Argentina | MID: 0.92% - NND: 1.84% |
|  | *Pimelodella sp1* |  |  |  |
|  | *Pimelodella sp.* |  |  |  |
| *Pimelodus albicans* | *Pimelodus sp.* | BOLD:AAZ5086 | Argentina | MID: 0.64% - NND: 2.41% |
| *Potamotrygon motoro* | *Potamotrygon cf. motoro* | BOLD:AAB5375 | Brazil | MID: 2.91% - NND: 1.69% |
|  | *Potamotrygon sp.* |  | Peru |  |
| *Prochilodus lineatus* | *Prochilodus sp.* | BOLD:AAB5650 | ND | MID: 3.68% - NND: 7.25% |
| *Pseudopimelodus mangurus* | *Pseudopimelodus sp.* | BOLD:AAD9623 | Brazil | MID: 1.42% - NND: 1.77% |
| *Pyrrhulina australis* | *Pyrrhulina sp.* | BOLD:ACG8000 | Argentina | MID: 0% - NND: 1.61% |
| *Rineloricaria parva* | *Rineloricaria cf. lima* | BOLD:AAZ4949 | Argentina | MID: 0.94 - NND: 6.21 |
|  | *Rineloricaria sp.* |  |  |  |
| *Serrasalmus marginatus* | *Serrasalmus aff. rhombeus* | BOLD:AAC7587 | Bolivia | MID: 1.96 - NND: 1.96 |
| *Brycon cf. hilarii* | *Brycon hilarii* | BOLD:AAE0479 | Brazil | MID: 0.16% - NND: 6.1% |
| *Crenicichla cf. lepidota* | *Crenicichla lepidota* | BOLD:ABZ6884 | Argentina | MID: 1.38% - NND: 1.67% |

*MI: Maximum Intraspecific , NN: Nearest Neighbour.

ND: No Data.
